# Dicyemid Mesozoans: A Unique Parasitic Lifestyle and a Reduced Genome

**DOI:** 10.1093/gbe/evz157

**Published:** 2019-07-26

**Authors:** Tsai-Ming Lu, Miyuki Kanda, Hidetaka Furuya, Noriyuki Satoh

**Affiliations:** 1 Marine Genomics Unit, Okinawa Institute of Science and Technology Graduate University, Japan; 2 DNA Sequencing Section, Okinawa Institute of Science and Technology Graduate University, Japan; 3 Department of Biology, Graduate School of Science, Osaka University, Japan; 4 Sars International Centre for Marine Molecular Biology, University of Bergen, Norway

**Keywords:** mesozoan, dicyemids, unique parasite, genome, reduction

## Abstract

Dicyemids, previously called “mesozoans” (intermediates between unicellular protozoans and multicellular metazoans), are an enigmatic animal group. They have a highly simplified adult body, comprising only ∼30 cells, and they have a unique parasitic lifestyle. Recently, dicyemids were shown to be spiralians, with affinities to the Platyhelminthes. In order to understand molecular mechanisms involved in evolution of this odd animal, we sequenced the genome of *Dicyema japonicum* and a reference transcriptome assembly using mixed-stage samples. The *D. japonicum* genome features a high proportion of repetitive sequences that account for 49% of the genome. The dicyemid genome is reduced to ∼67.5 Mb with 5,012 protein-coding genes. Only four Hox genes exist in the genome, with no clustering. Gene distribution in KEGG pathways shows that *D. japonicum* has fewer genes in most pathways. Instead of eliminating entire critical metabolic pathways, parasitic lineages likely simplify pathways by eliminating pathway-specific genes, while genes with fundamental functions may be retained in multiple pathways. In principle, parasites can stand to lose genes that are unnecessary, in order to conserve energy. However, whether retained genes in incomplete pathways serve intermediate functions and how parasites overcome the physiological needs served by lost genes, remain to be investigated in future studies.

## Introduction

**Fig. 1.**
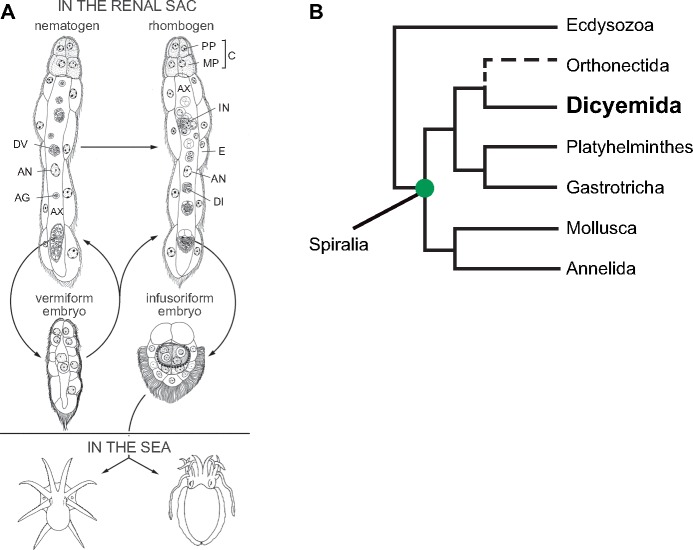
—The mesozoan, *Dicyema japonicum*, with a unique life cycle. (*A*) Life cycle of dicyemids. See the text for details. AG, agamete; AN, axial cell nucleus; AX, axial cell; C, calotte; DI, developing infusoriform embryo; DV, developing vermiform embryo; E, epidermal cell; IN, infusorigen; MP, metapolar cell; PP, propolar cell; Adapted and modified from Furuya and Tsuneki 2003. (*B*) Dicyemida is a member of Spiralia.

Dicyemids, together with orthonectids, were previously called “mesozoans,” an animal group of intermediate complexity between unicellular protozoans and multicellular metazoans (Stunkard 1954; Lapan and Morowitz 1972; Furuya and Tsuneki 2003; Brusca et al. 2016). Adults consist of only ∼30 cells (fig. 1*A*), and are parasitic in renal sacs of cephalopods. Their simplified bodies consist of three regions: a collate, a central axial cell, and ciliated epidermal cells (fig. 1*A*). The collate region (the most anterior eight cells) is used to attach to the surface of octopus renal tissues. The central axial cell is surrounded by an outer layer of ciliated epidermal cells that are used mainly for reproduction, where vermiform or infusoriform embryos develop. Ciliated epidermal cells absorb nutrients directly from host urine via endocytosis (Ridley 1968; Furuya and Tsuneki 2003). Dicyemids lack a digestive tract, coelom, circulatory system, and other differentiated tissues (fig. 1*A*). This is probably the most extreme case of secondary reduction of body plan complexity in spiralian parasites.

In contrast to the simple body plan, the life cycle of dicyemids is characterized by two reproductive modes (asexual and sexual) and there are larva and adult stages in each mode (fig. 1*A*). After infecting an octopus host, the germinal cell develops into an asexual reproductive adult nematogen (fig. 1*A*). Then, inside the central axial cell of the nematogen, the agamete (axoblast) develops into asexual reproductive vermiform embryos (Furuya et al. 2003) (fig. 1*A*). While embryos mature, the vermiform larvae escape from the axial cell of the nematogen and develop to new nematogens that attach to renal tissue of the same host, which increases the population density. Once the population density inside a host reaches a certain threshold or if nematogens receive certain chemical cues from the environment, nematogens transform into sexually reproductive adults, called rhombogens (fig. 1*A*) (Lapan and Morowitz 1972). Inside the central axial cell of a rhombogen, the hermaphroditic gonad (infusorigen) generates sperm and eggs. Released gametes fertilize and develop into mature, free-swimming infusoriform larvae (fig. 1*A*) (Furuya et al. 1992), which have to sense and locate new hosts in the open sea.

The phylogenetic position of this enigmatic group remained controversial for a long time (Giribet 2008; Edgecombe et al. 2011). Due to an extremely high rate of molecular changes in mesozoans, molecular phylogenetic approaches did not always produce reasonable conclusions (Katayama et al. 1995; Pawlowski et al. 1996; Petrov et al. 2010; Suzuki et al. 2010). A recent molecular phylogenetic analysis with sequence information for 348 nuclear genes showed that dicyemids have close affinities to orthonectids (fig. 1*B*) (Lu et al. 2017), although these have recently been categorized as two independent taxa (Stunkard 1954; Mikhailov et al. 2016). The Mesozoa has affinity for the Rouphozoa (Platyhelminths and Gastrotricha), rather than for mollusks and annelids (fig. 1*B*) (Lu et al. 2017). The possession of a “spiralian peptide” by dicyemids also supports the position that dicyemids are morphologically simplified spiralians (Kobayashi et al. 1999; Lu et al. 2017).

Parasitism is more common in animals than previously noted and has been reported in 15 of the 35 generally recognized animal phyla (Weinstein and Kuris 2016). Parasitism is likely to have evolved independently >200 times (Wang et al. 2011). Each parasitism event reflects the interaction of a given host–parasite pair, and adaptations to a parasitic lifestyle vary case by case. Parasites usually exhibit convergence, such as simplified morphology and complex lifecycles, reflecting selective pressures common to parasitism. Molecular convergence has also been reported across parasitic lineages, for example, in relation to gene expansions associated with parasite surface modifications to avoid triggering host immune systems (Wang et al. 2011). In addition, many unique adaptations have been observed at the genomic level that enable flatworm parasites to exploit specific niches (Zarowiecki and Berriman 2015). Thus, to understand evolution of parasitism, it is necessary to examine genomic innovations that make it possible. Comparative studies between parasites and closely related nonparasitic species could provide an opportunity to test long-standing hypotheses of genome reduction and molecular changes for adaptation to a parasitic lifestyle (Jackson 2015).

As mentioned earlier, dicyemids are microscopic endoparasites inhabiting renal sacs of cephalopods. Although more than one dicyemid species can inhabit an individual host (Furuya et al. 2003), dicyemids tend to be highly host-specific (Catalano 2013). Histological studies suggest that dicyemids employ endocytosis to absorb nutrients from host urine via epithelial cell membranes (Ridley 1969). However, there are many questions regarding the enigmatic lifestyle of dicyemids, especially, what genomic adaptations enable specific physiological functions of their parasitic lifestyle. To answer this question, we decoded the draft genome of the dicyemid, *Dicyema japonicum*. The dicyemid genome not only reveals secrets of dicyemid biology but also offers insights into evolution of parasitism.

## Materials and Methods

### Biological Materials

For genome sequencing, *D.japonicum* specimens were collected from a single, adult *Octopus sinensis*. Specimens of mixed adult and larval stages were separated from the octopus renal sac tissue. They were rinsed with filtered seawater several times to remove octopus cells. These samples were frozen and stored at −20 °C. Further, pooled dicyemid specimens were also isolated from seven octopuses for RNA extraction. Because complete removal of octopus cells was infeasible, the gonad of a male *O. sinensis* was dissected for genome sequencing, and was used as a reference to remove host sequence contamination.

### Genome Sequencing

**Fig. 2.**
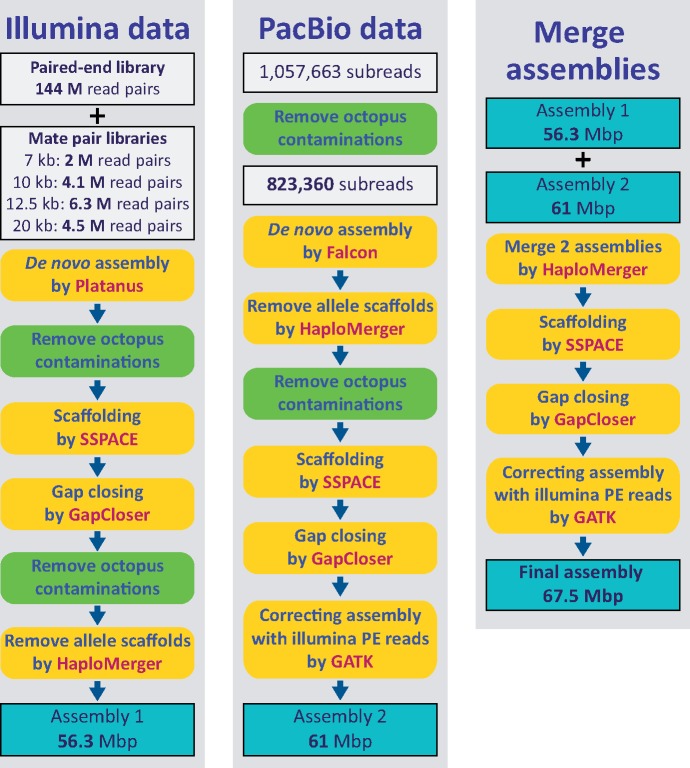
—A pipeline for sequencing and de novo assembly of the *Dicyema japonicum* genome. Sequencing data generated by Illumina and PacBio sequencing platforms were first assembled separately. Then the two data sets were merged into a final assembly for downstream analyses. See Materials and Methods for details.

The strategy of genome sequencing and assembly employed is shown in figure 2 and supplementary table S1, Supplementary Material online. Genomic DNA was extracted from samples using Promega Maxwell 16 Systems and Maxwell 16 Cell DNA purification kits (Promega, No. AS1020). A dicyemid paired-end library with insert sizes of 600 bp was prepared using TruSeq DNA PCR-Free Library Prep Kits (Illumina, No. 20015962), and sequenced on an Illumina MiSeq. Four mate-pair libraries of insert lengths (1.6–7, 7–10, 10–12.5, and 12.5–20 kb) were prepared using a Nextera Mate Pair Sample Preparation Kit (Illumina, No. FC-132-1001), and sequenced on an Illumina HiSeq 2500. In addition, PacBio extra-long reads were generated using the single-molecule real-time sequencing method on a PacBio RS II (supplementary table S1, Supplementary Material online). The octopus-genome paired-end library was sequenced using rapid run mode on an Illumina HiSeq 2500 (supplementary table S1, Supplementary Material online).

### The Contamination-Removing Assembly Pipeline

Eight runs of MiSeq sequencing generated 207 million read pairs of paired-end library (supplementary table S1, Supplementary Material online). The quality-trimming process employed Trimmomatic (v0.33) (Bolger et al. 2014) with a quality threshold of 20 and a minimum length of 50 bases (SLIDINGWINDOW: 4: 20, LEADING: 20, TRAILING: 20, MINLEN: 50). In addition, the sequencing of four mate-pair libraries generated 76.3 million read pairs (supplementary table S1, Supplementary Material online). NextClip (Leggett et al. 2014) was used to categorize mate-pair reads by checking the presence of adaptors on both reads. Reads without the junction adaptor likely arose from paired-end sequences that slipped through the biotin enrichment process and they were discarded. After NextClip-filtering and quality-trimming, 22% of mate-pair reads were used for scaffolding. We also obtained sequences with lengths up to 65 kb from the PacBio platform, and 86% of them were retained after the decontamination process. They were employed in de novo assembling and scaffolding. In our genome assembly pipeline (fig. 2), we first assembled de novo the Illumina and PacBio data separately using Platanus (v1.2.4) (Kajitani et al. 2014) and Falcon (Chin et al. 2016), respectively. Then, we merged the two preliminary assemblies into the final assembly for further analyses.

To eliminate possible octopus sequences from the assembly, output sequences from Platanus were mapped using 562 million octopus Illumina paired-end reads. They were deleted if the average mapped base coverage exceeded 1. The remaining Platanus assembly was then scaffolded with SSPACE (Boetzer et al. 2011) and SSPACE-LongRead (Boetzer and Pirovano 2014), incorporating sequences of four mate-pair libraries and PacBio long-read sequences, respectively. Then gap closing was performed using GapCloser (Luo et al. 2012) with all paired-end library reads. Before removing redundant allelic scaffolds to obtain a haploid genome assembly with HaploMerger (Huang et al. 2012), the decontamination filtering process using octopus read-mapping was performed again to obtain the final genome assembly.

### Genome Size and Heterozygosity Estimation

We employed *k*-mer coverage-based methods to estimate genome size using paired-end reads from two MiSeq runs. Jellyfish (v.2.1.3) (Marcais and Kingsford 2011) was used to count *k*-mer occurrences of paired-end reads with a setting of *k *=* *17 (supplementary fig. S1, Supplementary Material online). Plotting a histogram of *k*-mer distributions, peak coverage was supposed to be the average *k-*mer coverage. Assuming that the average *k*-mer coverage is equal to the total number of *k*-mers divided by the genome size, we estimated the genome size. The 17-mer profile was also used to estimate overall characteristics, including genome size and heterozygosity with an open-source tool GenomeScope (Vurture et al. 2017), which applies a mixture model of four evenly spaced, negative binomial distributions to the *k*-mer profile. The GenomeScope analysis indicated that the estimated genome size of *D.japonicum* is ∼65 Mb with a heterozygosity rate of 1.24%.

### Transcriptome Sequencing, Assembly, and Annotation

RNA was extracted from pooled dicyemid specimens isolated from seven octopuses using a Direct-zol RNA MicroPrep Kit (Zymo Research, No. R2060). A stranded library was prepared using a NEBNext Ultra Directional RNA Library Prep Kit for Illumina (NEB, No. E7420), and sequencing was performed on an Illumina HiSeq4000.

Raw reads were quality filtered (*Q* score ≥20) and trimmed with Trimmomatic (v0.33). Afterward, quality-trimmed reads were assembled de novo using Trinity (v2.0.6) (Grabherr et al. 2011) with default settings. TransDecoder was utilized to extract coding regions and to translate transcripts into amino acid sequences (Haas et al. 2013). Although dicyemid samples were washed with filtered seawater several times, we still could not preclude the possibility of octopus cell contamination. We performed an assessment to confirm that the dicyemid transcriptome assembly was uncontaminated. We mapped 562 million octopus paired-end reads back to the dicyemid transcriptome assembly using Bowtie 2 (v2.2.3) (Langmead and Salzberg 2012). Only 1% of the transcripts were mapped to octopus reads, which were removed from the data.

### Gene Modeling

Before predicting genes, the gene predictor AUGUSTUS (Keller et al. 2011) was trained with a dicyemid training gene set to obtain dicyemid-specific prediction parameters. In order to create a training gene set, a PASA alignment assembly was first generated according to the genome assembly, as well as Trinity-assembled cDNA sequences, and removal of redundant sequences with CD-HIT (Fu et al. 2012) using a 95% identity threshold. Protein coding regions of the PASA alignment assembly were extracted, and then redundant (>80% identity) and possibly error-causing genes were filtered. The training gene-set was randomly divided into two parts. A test set of 300 genes was used to evaluate prediction accuracy of training parameters, and the remaining genes were applied for training. A script autoAugTrain.pl in AUGUSTUS package was trained with the training gene set, and optimize_augustus.pl performed ≤5 rounds of optimization to acquire dicyemid-specific parameters for gene prediction.

Repeated sequences should be masked prior to mapping of transcriptomic data to generate evidence of exons, and later gene prediction should be run on the unmasked genome using evidence generated from repeat regions. RepeatScout (Price et al. 2005) was used to discover repetitive DNA regions and to count the frequency of these regions, creating an index. Then, RepeatMasker (Smit et al. 2013–2015) was used to mask repeat regions in the genome when repeated sequences occurred >30 times. Coordinate position information for 140,794 repeat regions in the genome was obtained from RepeatMasker output. cDNA sequences of Trinity and PASA assemblies were aligned against the masked genome with BLAT (Kent 2002) with at least 80% identity. The blat2hints.pl script used Trinity alignments and PASA assemblies to indicate cDNA sequences. We then incorporated quality-trimmed transcriptomic reads in a two-step iterative mapping approach to generate suggestions for exons and introns. In the first step, spliced-alignments were performed with Tophat (Kim et al. 2013) to identify introns, and that evidence was used to predict genes with AUGUSTUS. By concatenating data regarding introns and predicted genes from the first step, we created a database of exon–exon junctions. The second step was to map quality-trimmed transcriptomic reads against exon–exon junctions with Bowtie (Langmead and Salzberg 2012), which increased the number of reads aligned to splice sites. Then, new evidence generated from the merger of second-round alignments increased gene prediction accuracy. Last, gene models were predicted by referring to pretrained parameters and evidence for introns, exons, and repeats.

Functional domains were identified by Pfam domain search (Finn et al. 2016) using HMMER (v3.1b2) (Mistry et al. 2013), and results were selected with e-values threshold of 1e^−5^. A custom script was used to count how many genes contain specific domains. For pathway analysis, we adopted the online KEGG Automatic Annotation Server to assign each predicted gene to a KEGG ortholog using the bidirectional best hit method and to map assigned orthologs to KEGG reference pathways. Amino acid sequences of selected bilaterian species (supplementary table S2, Supplementary Material online) were employed for clustering orthologous groups using OrthoMCL (Fischer et al. 2011). Predicted gene models were annotated with reciprocal BLASTP searches against the Swiss-Prot database downloaded from UniProt (Boutet et al. 2016). Search output was filtered with the e-value threshold of 1e^−5^.

### Clustering of Orthologous Groups

OrthoMCL was utilized to cluster orthologous groups among selected bilaterian species (supplementary table S2, Supplementary Material online). Low-quality gene model sequences were removed based on the sequence length of OrthoMCL criteria. Sequences were applied to all-versus-all BLAST searches with e-value cutoff of 1e^−5^. The results proceeded through the internal algorithm of OrthoMCL to separate protein pairs into three relationship categories, namely orthologs, in-paralogs, and co-orthologs. Then MCL (Enright et al. 2002) clustered the pairs into final orthologous groups and singletons that were not assigned into any orthologous group. The Venn diagram of shared orthologous groups between dicyemids, orthonectids, and other spiralians was plotted using jvenn online service (Bardou et al. 2014). A custom Perl script was used to count the number of predicted genes in each ortholog group from each species.

### Annotation of Hox Genes

To search for Hox cluster genes, 16 candidate gene models containing homeobox domains were used as queries in reciprocal BLASTP searches against the Homeobox Database (Zhong and Holland 2011) and the Swiss-Prot database. Candidate gene models that did not have bidirectional best hits were submitted manually to NCBI BLASTP searches against the nr database. Afterward, putative Hox genes from BLAST searches were identified by phylogenetic analysis inferred from homeobox domain amino-acid sequences. Sequences of *Lottia*, *Capitella*, *Drosophila*, and *Branchiostoma* were retrieved from the supplemental database of Simakov et al. (2013) and the Homeobox Database. The data set was aligned using MAFFT (v7.220) (Katoh and Standley 2013) and trimmed using trimAl (v1.2) (Capella-Gutierrez et al. 2009). A gene tree was reconstructed using RAxML (v8.1.20) (Stamatakis 2014) based on the maximum likelihood method under the LG substitution model and the GAMMA model of rate heterogeneity with 1,000 bootstrap replications.

The Hox cluster has been used to assess the completeness and continuity of genome assembly (Shields et al. 2018). However, dicyemids retain only four Hox genes with a disorganized cluster structure. On the other hand, most protein-coding sequences of nonredundant transcripts (91.9%) from a mixed-stage sample could be mapped to the current genome assembly using GMAP (Wu and Watanabe 2005), with the criteria of query coverage and identity >90% (–min-identity = 0.9 –min-trimmed-coverage = 0.9), implying that the current genome assembly covers most expressed genes. In addition, the conspicuous reduction of Hox genes has been reported in other parasites, such as orthonectids (Mikhailov et al. 2016) and tapeworms (Tsai et al. 2013), which may reflect convergent simplification of parasitic body organization. Moreover, *Paedocypris* fishes also show extensive Hox gene loss as an apparent adaptation to an extreme habitat (Pándy-Szekeres et al. 2018). These studies raise doubts about whether the Hox cluster is a proper indicator of genome assembly completeness and contiguity, particularly for parasites.

## Results and Discussion

### The *Dicyema* Genome Is Highly Reduced

**Fig. 3.**
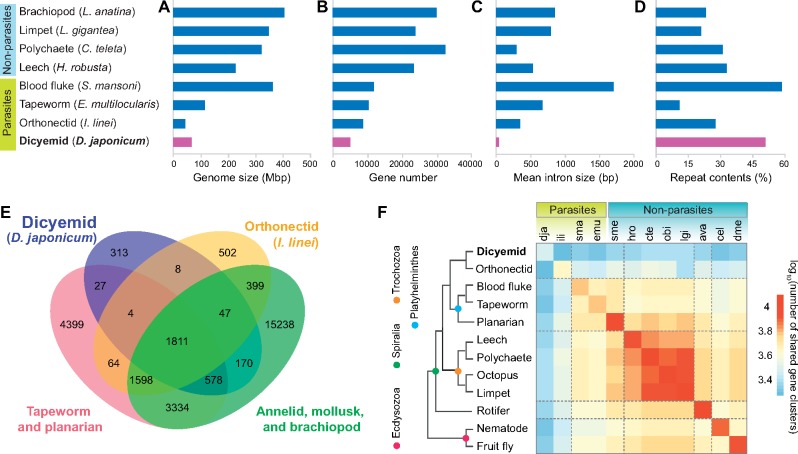
—Genomic and genic reduction in *Dicyema japonicum.* (*A–D*) Comparisons of genomic characteristics among nonparasitic and parasitic spiralians. (*A*) The genome size, (*B*) gene number, (*C*) intron size, and (*D*) percentage of repeats in the genome. Genomic information of compared taxa was obtained from published sources: brachiopods (Luo et al. 2015); limpets, polychaetes, and leeches (Simakov et al. 2013); blood flukes (Berriman et al. 2009); tapeworms (Berriman et al. 2009); and orthonectids (Mikhailov et al. 2016). (*E*) Venn diagram of dicyemid orthologous groups shared with other spiralians. Gene models of *D. japonicum* present 2,958 gene clusters, including 313 lineage-specific clusters. (*F*) Matrix of shared gene clusters among selected spiralians and ecdysozoans. Dicyemids share fewer gene clusters with orthonectids than with platyhelminths and lophotrochozoans. In general, parasitic spiralians share fewer gene clusters with nonparasitic spiralians.

The haploid genome of *D.japonicum* is 67.5 Mb, the assembly consisting of 377 scaffolds with an N50 size of 1 Mb, close to the estimated genome size of 65 Mb (table 1, fig. 3*A*, andsupplementary fig. S1, Supplementary Material online). The genome exhibits high heterozygosity (1.24%). We predicted 5,012 protein-coding genes in the *Dicyema* genome (table 1), and 92% of nonredundant transcripts from mixed-stage specimens were mapped to the genome assembly. CEGMA and BUSCO tests demonstrated that 77% and 76% of conserved core eukaryotic genes were identified in the *Dicyema* genome assembly, respectively.

**Table 1:** Summary of Genome Assembly

Genome size (Mb)	67.5
GC content (%)	36.7
Gap rate (%)	4.1
Repeats (%)	48.9
Number of contigs	1,965
Number of scaffolds	377
Contigs per scaffold	5.2
Contig N50 (kb)	195.9
Scaffold N50 (kb)	1,000.2
Number of predicted genes	5,012
Mean coding seq. size (bp)	1,155.2
Introns per gene	6.2
Exons per gene	7.6
Mean intron size (bp)	38.2
Mean exon size (bp)	198.2

Except for the blood fluke, *Schistosoma mansoni*, parasites, including the dicyemid, *D. japonicum*, the tapeworm, *Echinococcus multilocularis*, and the orhthonecid, *Intoshia linei*, have reduced genomes (fig. 3*A*), gene numbers (fig. 3*B*), and intron sizes (fig. 3*C*). These were most conspicuous in *D. japonicum*, most notably in the average intron size of 38 bp (fig. 3*C*). The *D. japonicum* genome also features a high proportion of repetitive sequences that account for 49% of the genome (table 1, fig. 3*D*, andsupplementary fig. S2, Supplementary Material online). Multiple copies of LINE (long interspersed nuclear element)-1 retrotransposable-element ORF2 protein could amplify retrotransposons (supplementary table S3, Supplementary Material online), which might lead to larger numbers of repetitive sequences in the dicyemid genome (supplementary fig. S2, Supplementary Material online).

### Genetic Characters of the *Dicyema* Genome

In relation to the reduced gene number in the *D. japonicum* genome, using OrthoMCL (Fischer et al. 2011) we examined how many and what kinds of orthologous gene clusters are shared among 16 protostome species (supplementary table S2, Supplementary Material online). We found that while 1,811 clusters are shared by all spiralians analyzed, *D. japonicum* contained 2,958 clusters with 313 that are lineage-specific (fig. 3*E*). In general, parasitic spiralians share fewer gene clusters than nonparasitic spiralians, and dicyemids and orthonectids share even fewer than platyhelminths and lophotrochozoans (fig. 3*F*). This suggests that the reductive evolution of parasitic lineages has resulted in different genic architectures.

In relation to their unique lifestyle, *D. japonicum* exhibits ten well-characterized gene clusters with more than six copies apiece (supplementary table S3, Supplementary Material online). The top six are potentially associated with endocytosis, participating in membrane invagination, formation of new vesicles, and movement of endosomes from the plasma membrane into the cytosol. For example, the actin cytoskeleton could be involved in each endocytic step (Qualmann et al. 2000) and the dynein motor complex contributes to movement of endosomes (Flores-Rodriguez et al. 2011). Macrophage mannose receptor has been reported to mediate cellular uptake and endosomal delivery of various molecules, such as lipoglycans, oligodeoxynucleotides, and metalloproteinases (Prigozy et al. 1997; Sorvillo et al. 2012; Moseman et al. 2013). Similarly, MFS (major transporter superfamily) SLC46A3 participates in intracellular transport (Bissa et al. 2016). Vitellogenic carboxypeptidase may be involved in actin remodeling in membrane ruffles (Harris et al. 2006). Expansion of these gene clusters may facilitate nutrient-uptake through membrane ruffles on dicyemid epithelial cells from the host. In addition to its endocytotic function, actin is also associated with cilia beating to generate currents that circulate urine in the renal sac and continuously bring nutrients to the dicyemid surface for endocytosis. Furthermore, cilia on infusoriform larvae likely contribute to efficient mobility in open seawater to approach new hosts. These multicopy genes explain how dicyemids adapt physiologically to their unique lifestyle.

### Genetic Background of Highly Simplified Body Architecture

Functional domains contribute structural characteristics and specific functions to proteins. Comparisons of Pfam domain searches among bilaterians showed that parasitic spiralians contain fewer functional domain-containing genes in general (supplementary table S4, Supplementary Material online). Reflecting their highly simplified body organization, dicyemids possess the fewest functional domain-containing genes of transcription factors and signaling molecules (supplementary tables S5 and S6, Supplementary Material online). The fibroblast growth factor (Fürthauer M et al. 2004), Hedgehog N-terminal signaling (Yoshino et al. 2016), and CHRD domain of *Chordin* gene (Saina et al. 2009) that regulate body organization patterning in early embryos were absent (supplementary tables S5 and S6, Supplementary Material online). TGFβ and Wnt signaling pathways are essentially involved in axis formation and cell fate specification during embryonic development (Massagué 2012; Niehrs 2012). However, parasitic spiralians (dicyemid, orthonectid, tapeworm, and blood fluke) all possess less than half the number of TGFβ and Wnt domain-containing genes of nonparasitic spiralians, and only one Wnt family domain-containing gene was found in *D. japonicum* (supplementary table S6, Supplementary Material online). The loss of Wnt genes has also been reported in parasitic flatworms (Riddiford and Olson 2011). In terms of nervous system development, neuronal helix-loop-helix transcription factor and Delta serrate ligand domains, which are active in neuron cell fate determination, are missing as well (supplementary table S5, Supplementary Material online).

**Fig. 4.**
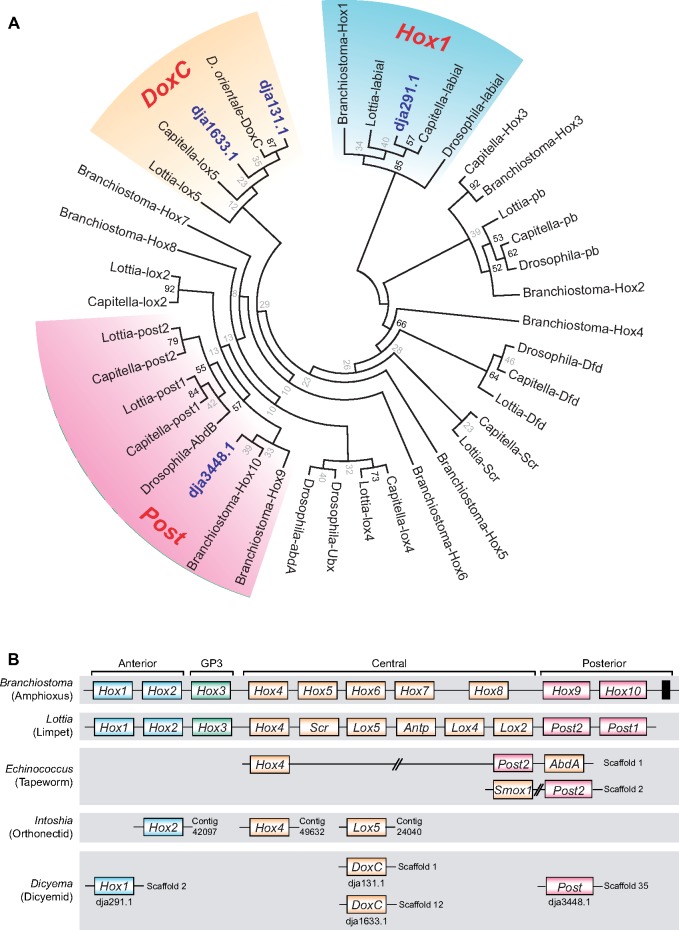
—*Dicyema japonicum* contains four putative unclustered Hox genes. (*A*) A phylogenetic tree inferred from the homeobox domain using the maximum likelihood method, showing the presence of four Hox genes (blue bolds) in the *D. japonicum* genome. (*B*) Hox gene synteny in selected bilaterians. The scattered, unclustered Hox gene structure occurs in three parasitic spiralian lineages. The black block in *Branchiostoma* represents the rest of the posterior Hox genes. Double slashes signify noncontinuous linkage between two genes.

Only 16 homeobox domain-containing genes were found in the dicyemid genome, which is about one-fourth the number found in other parasitic spiralians (supplementary table S5, Supplementary Material online). Conserved developmental transcription factors encoded by Hox genes help to govern anterior–posterior (A–P) pattering in diverse bilaterians. In *D. japonicum*, only four putative Hox genes were annotated and further characterized by phylogenetic analysis (fig. 4*A*). Two putative dicyemid Hox genes are *Hox1* and *Post*-like genes, and they could be representative anterior and posterior Hox genes, respectively. The other two are *Lox5*-like genes, which could represent central Hox genes. One of them was recognized as the previously published dicyemid *DoxC* gene (Kobayashi et al. 1999). Because the *Lox4/5-*like gene is specific to lophotrochozoans, the presence of these genes in the *Dicyema* genome provides further evidence that they are indeed members of that superphylum.

However, these four genes were not located on a single scaffold; thus, a Hox cluster was not observed in the *D. japonicum* genome (fig. 4*B*). Similarly, orthonectids, another parasitic lineage with close affinity to dicyemids (Lu et al. 2017), retain one anterior (*Hox2*) and two central Hox genes (*Hox4* and *Lox5*) (Mikhailov et al. 2016). Although both dicyemids and orthonectids have no specialized segmentation, these Hox gene sets seem sufficient to govern basic A–P polarity. In addition, the conspicuous reduction of Hox cluster genes in parasites (dicyemids, orthonectids, Mikhailov et al. 2016; tapeworms, Tsai et al. 2013; and flatworms, Olson 2008) may reflect convergent simplification of parasite morphological organization (fig. 4*B*).

### Convergent Reduction of the Number of Genes in Functional Pathways for Parasites

**Fig. 5.**
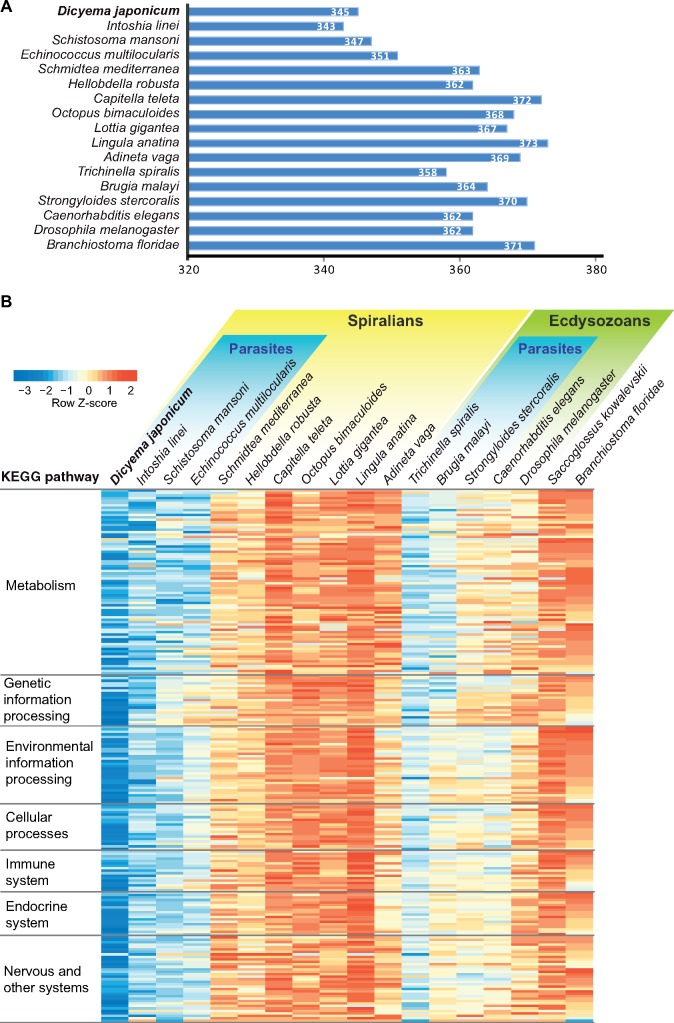
—Reduction of genes associated with biological pathways in parasites. (*A*) Numbers of conserved pathways in bilaterian species. (*B*) A heatmap showing the number of genes involved in conserved pathways to compare them among selected bilaterian species. Although parasitic lineages possess only 5% fewer conserved KEGG pathways than nonparasitic lineages (*A*), parasites in general have fewer genes in each of the KEGG pathways than nonparasitic species (*B*). *Dicyema japonicum* exhibits the fewest genes among all parasites.

We mapped dicyemid genes to KEGG reference pathways, which were compared with 18 bilaterians, including 7 parasitic lineages, and 11 nonparasitic lineages among the Spiralia, Ecdysozoa, and Deuterostomia. *Dicyemajaponicum* genes exist in 345 KEGG reference pathways, which is only 5% less than the average number of pathways (362) among other bilaterians (fig. 5*A*). This might create the impression that *D. japonicum* retains most functional bilaterian pathways, despite their extreme simplification in morphological traits or lifestyle. In fact, KAAS (KEGG Automatic Annotation Server) annotated 2111 KEGG orthologs from dicyemid gene models, and 688 of them are involved in two or more KEGG pathways. Thus, it is likely that some pathways of dicyemids lost genes, in some cases, retaining only a few genes. Therefore, the presence of some genes in certain pathways does not mean that those pathways are fully functional. Owing to differing degrees of pleiotropy among genes in a given pathway, some that play more fundamental roles may be involved in multiple pathways that are physiologically crucial for dicyemid survival.

In contrast to comparable numbers of biological pathways among bilaterians, both spiralian and ecdysozoan parasites possess fewer genes in most pathways than nonparasites, particularly in metabolic pathways, as reported in flukes and tapeworms (Wang et al. 2011; Tsai et al. 2013) (fig. 5*B* andsupplementary fig. S3, Supplementary Material online). Because *D. japonicum* possesses far fewer genes than other bilaterians (fig. 3*B*), we further examined gene distribution in KEGG pathways. We found that *D. japonicum* has fewer genes in most pathways (fig. 5*B* andsupplementary fig. S3, Supplementary Material online). Instead of eliminating entire metabolic pathways, parasitic lineages likely simplify pathways by eliminating pathway-specific genes, but they retain genes that participate in multiple pathways and that have fundamental functions. In principle, parasites may delete genes that are useless in a parasitic lifestyle, in order to conserve energy. However, whether retained genes in incomplete pathways serve intermediate functions and how parasites overcome the physiological gaps caused by lost genes, remain to be investigated in future studies. 

## Supplementary Material

Supplementary data are available at *Genome Biology and Evolution* online.

## Supplementary Material

evz157_Supplementary_Data

## Notes

**Data deposition:** This genome project has been registered at NCBI under BioProject accession PRJNA496385. Sequencing reads of the genome and transcriptomes have been deposited at the NCBI Sequence Read Archive (SRR8079359, SRR8079349-52, SRR8079348, SRR8079591, SRR8061618, SRR8061630-3). Genome and transcriptome assemblies have been deposited at DDBJ/ENA/GenBank (RDEW00000000) and NCBI Transcriptome Shotgun Assembly Sequence Database (GHAB00000000), respectively. A genome browser is available at https://marinegenomics.oist.jp.
