# Supplementary material for: Dicyemid Mesozoans: A Unique Parasitic Lifestyle and a Reduced Genome
Source: Genome Biol Evol. 2019 Jul 26;11(8):2232–43. doi: 10.1093/gbe/evz157 (PMC6736024; doi:10.1093/gbe/evz157)
Supplement: evz157_Supplementary_Data [file evz157_supplementary_data.zip › Fig_S3.pdf]

Figure S3.

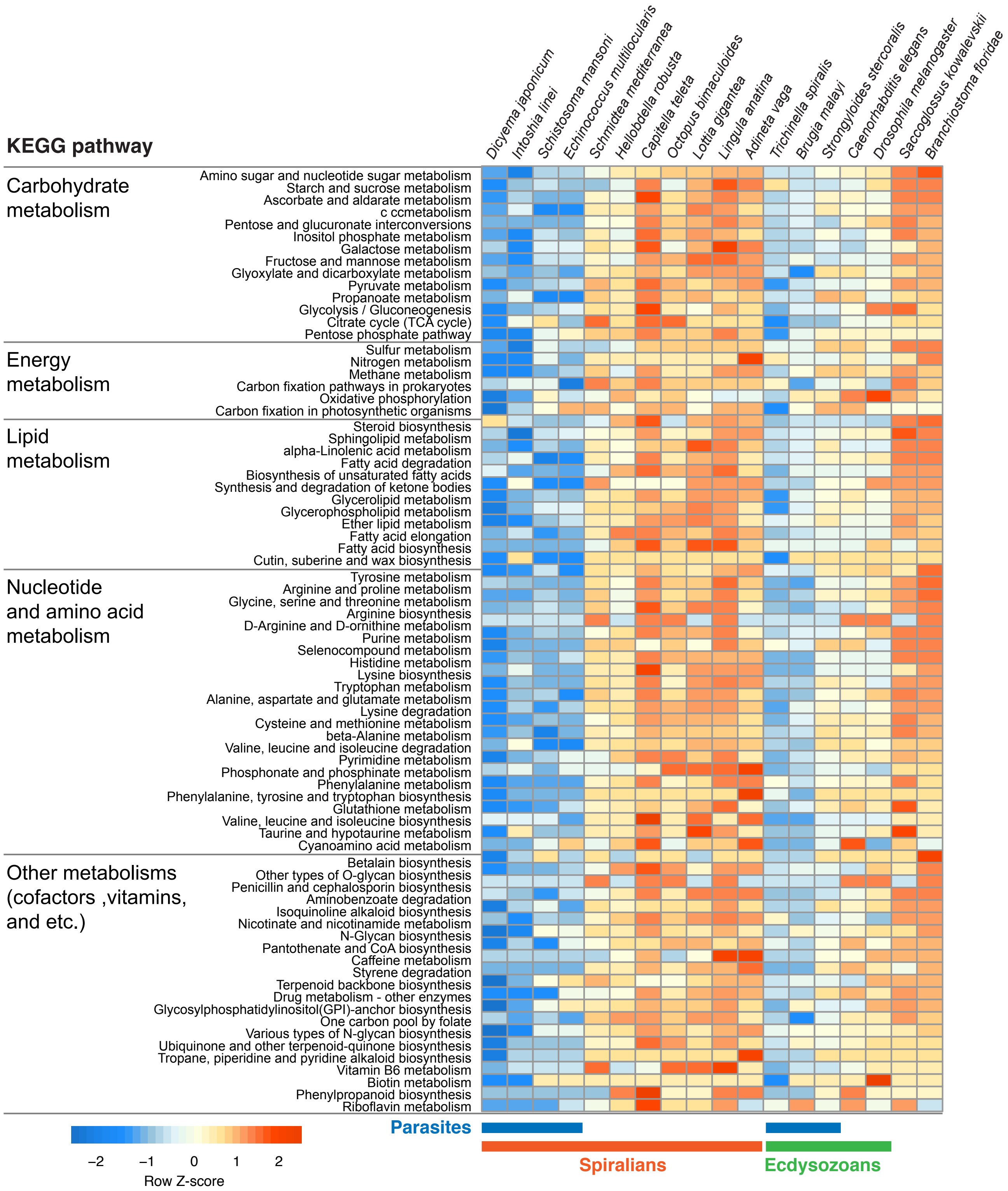
KEGG pathway
Dicyema japonicum
Intoshia linei
Schistosoma mansoni
Echinococcus multilocularis
Schmidtea mediterranea
Helobdella robusta
Capitella teleta
Octopus bimaculoides
Lottia gigantea
Lingula anatina
Adineta vaga
Trichinella spiralis
Brugia malayi
Strongyloides stercoralis
Caenorhabditis elegans
Drosophila melanogaster
Saccoglossus kowalevskii
Branchiostoma floridae
Carbohydrate metabolism
Amino sugar and nucleotide sugar metabolism
Starch and sucrose metabolism
Ascorbate and aldarate metabolism
c ccmetabolism
Pentose and glucuronate interconversions
Inositol phosphate metabolism
Galactose metabolism
Fructose and mannose metabolism
Glyoxylate and dicarboxylate metabolism
Pyruvate metabolism
Propanoate metabolism
Glycolysis / Gluconeogenesis
Citrate cycle (TCA cycle)
Pentose phosphate pathway
Energy metabolism
Sulfur metabolism
Nitrogen metabolism
Methane metabolism
Carbon fixation pathways in prokaryotes
Oxidative phosphorylation
Carbon fixation in photosynthetic organisms
Lipid metabolism
Steroid biosynthesis
Sphingolipid metabolism
alpha-Linolenic acid metabolism
Fatty acid degradation
Biosynthesis of unsaturated fatty acids
Synthesis and degradation of ketone bodies
Glycerolipid metabolism
Glycerophospholipid metabolism
Ether lipid metabolism
Fatty acid elongation
Fatty acid biosynthesis
Cutin, suberine and wax biosynthesis
Nucleotide and amino acid metabolism
Tyrosine metabolism
Arginine and proline metabolism
Glycine, serine and threonine metabolism
Arginine biosynthesis
D-Arginine and D-ornithine metabolism
Purine metabolism
Selenocompound metabolism
Histidine metabolism
Lysine biosynthesis
Tryptophan metabolism
Alanine, aspartate and glutamate metabolism
Lysine degradation
Cysteine and methionine metabolism
beta-Alanine metabolism
Valine, leucine and isoleucine degradation
Pyrimidine metabolism
Phosphonate and phosphinate metabolism
Phenylalanine metabolism
Phenylalanine, tyrosine and tryptophan biosynthesis
Glutathione metabolism
Valine, leucine and isoleucine biosynthesis
Taurine and hypotaurine metabolism
Cyanoamino acid metabolism
Other metabolisms (cofactors ,vitamins, and etc.)
Betalain biosynthesis
Other types of O-glycan biosynthesis
Penicillin and cephalosporin biosynthesis
Aminobenzoate degradation
Isoquinoline alkaloid biosynthesis
Nicotinate and nicotinamide metabolism
N-Glycan biosynthesis
Pantothenate and CoA biosynthesis
Caffeine metabolism
Styrene degradation
Terpenoid backbone biosynthesis
Drug metabolism - other enzymes
Glycosylphosphatidylinositol(GPI)-anchor biosynthesis
One carbon pool by folate
Various types of N-glycan biosynthesis
Ubiquinone and other terpenoid-quinone biosynthesis
Tropane, piperidine and pyridine alkaloid biosynthesis
Vitamin B6 metabolism
Biotin metabolism
Phenylpropanoid biosynthesis
Riboflavin metabolism
−2
−1
0
1
2
Row Z-score
Parasites
Spiralians
Ecdysozoans
