# Supplementary material for: Dicyemid Mesozoans: A Unique Parasitic Lifestyle and a Reduced Genome
Source: Genome Biol Evol. 2019 Jul 26;11(8):2232–43. doi: 10.1093/gbe/evz157 (PMC6736024; doi:10.1093/gbe/evz157)
Supplement: evz157_Supplementary_Data [file evz157_supplementary_data.zip › Legends of Supplementary Figures.docx]

**Legends of Supplementary Figures**

**Supplementary Fig. 1. The *Dicyema* genome is compact and exhibits high heterozygosity.** The GenomeScope profile shows that homozygous peak coverage (arrow) is twice as great as heterozygous peak coverage (arrowhead), and the error rate was 0.012% after error correction using the Corrector AR program in SOAPec. The estimated genome size of *D. japonicum* is about 65 Mbp with a heterozygosity rate of 1.24%.

**Supplementary Fig. 2.** Distribution of repetitive sequences in the *Dicyema japonicum* genome.

**Supplementary Fig. 3. Parasites possess fewer genes in metabolic pathways.**

The heatmap of conserved metabolic pathways in selected bilaterians showing that not only in spiralians, but also among ecdysozoans, except for *Strongyloides stercoralis*, parasitic species retain far fewer genes in metabolic pathways than non-parasites. This suggests convergent evolution of parasites, at least in spiralian lineages.
