# Supplementary material for: Dicyemid Mesozoans: A Unique Parasitic Lifestyle and a Reduced Genome
Source: Genome Biol Evol. 2019 Jul 26;11(8):2232–43. doi: 10.1093/gbe/evz157 (PMC6736024; doi:10.1093/gbe/evz157)
Supplement: evz157_Supplementary_Data [file evz157_supplementary_data.zip › Suppl Tables.docx]

**Supplemental Tables**

| **Table S1. Summary of dicyemid genome sequencing data** | | | | | | | |
| --- | --- | --- | --- | --- | --- | --- | --- |
| Sequencing platform | Method | Library Length | Read Length | | Raw read pairs | | Raw bases |
| *Dicyema japonicum* genome | |  |  | |  | |  |
| Illumina MiSeq | Paired-end | 600 | 2 x 300 | | 206,959,118 | | 113,349,153,168 |
| Illumina HiSeq | Mate pair (Nextera) | 1,600-7,000 | 2 x 150 | | 4,433,810 | | 982,878,355 |
| Illumina HiSeq | Mate pair (Nextera) | 7,000-10,000 | 2 x 150 | | 8,683,880 | | 1,919,579,646 |
| Illumina HiSeq | Mate pair (Nextera) | 10,000-12,500 | 2 x 150 | | 12,629,929 | | 2,790,166,864 |
| Illumina HiSeq | Mate pair (Nextera) | 12,500-20,000 | 2 x 150 | | 9,097,714 | | 2,003,644,168 |
| PacBio RS II | SMRT | >7,000 | 7,068^a^ | | 1,057,663^b^ | | 6,797,850,486 |
| *Octopus sinensis* genome | |  |  | |  |  | |
| Illumina HiSeq | Paired-end | 600 | 2 x 250 | 312,232,334 | | 181,116,326,462 | |
| ^a^ PacBio subread mean length; ^b^ subread number by PacBio | | | |  | |  | |

| **Table S2. Selected bilaterian species for comparative analyses** | | | | |
| --- | --- | --- | --- | --- |
| Short ID | Scientific name | Common name | Gene number | Download source |
| Spiralians |  |  |  |  |
| dja | *Dicyema japonicum* | Dicyemid | 5,012 | This study |
| ili | *Intoshia linei* | Orthonectid | 8,724 | NCBI |
| sma | *Schistosoma mansoni* | Blood fluke | 11,723 | UniProt |
| emu | *Echinococcus multilocularis* | Tapeworm | 10,656 | WormBase |
| sme | *Schmidtea mediterranea* | Planarian | 32,615 | SmedGD |
| hro | *Helobdella robusta* | Leech | 23,328 | Uniprot |
| cte | *Capitella teleta* | Polychaete | 31,236 | Uniprot |
| obi | *Octopus bimaculoides* | Octopus | 38,585 | OIST |
| lgi | *Lottia gigantea* | Limpet | 23,721 | Uniprot |
| lan | *Lingula anatina* | Brachiopod | 29,907 | OIST |
| ava | *Adineta vaga* | Rotifer | 49,300 | Genoscope |
| Ecdysozoans | |  |  |  |
| tsp | *Trichinella spiralis* | Nematode (Clade I) | 16,380 | WormBase |
| bma | *Brugia malayi* | Nematode (Clade III) | 13,436 | WormBase |
| sst | *Strongyloides stercoralis* | Nematode (Clade IV) | 13,098 | WormBase |
| cel | *Caenorhabditis elegans* | Nematode (Clade V) | 25,847 | Uniprot |
| dme | *Drosophila melanogaster* | Fruit fly | 19,684 | Uniprot |
| Deuterostomes | |  |  |  |
| sko | *Saccoglossus kowalevskii* | Acorn worm | 22,111 | OIST |
| bfl | *Branchiostoma floridae* | Amphioxus | 28,544 | Uniprot |
| has | *Homo sapiens* | Human | 20,257 | Uniprot |

| **Table S3. Ten characterized gene clusters of *Dicyema japonicum*** | | |
| --- | --- | --- |
| Gene cluster | Copy number | Annotation |
| OG_2^a^ | 16 | Dynein heavy chain 1 |
| OG_10592^a^ | 15^b^ | Macrophage mannose receptor |
| OG_52^a^ | 9 | Actin, cytoplasmic A3 |
| OG_1661^a^ | 9^b^ | Vitellogenic carboxypeptidase-like protein (CPVL) |
| OG_8859^a^ | 7^b^ | Conserved oligomeric Golgi complex subunit 8 (COG8) |
| OG_18681^a^ | 7 | MFS transporter (SLC46A3) |
| OG_2865 | 6^b^ | Malonyl-CoA-acyl carrier protein transacylase |
| OG_6419 | 6^b^ | LINE-1 retrotransposable element ORF2 protein |
| OG_20785^a^ | 6 | Rho GTPase-activating protein |
| OG_20788 | 6 | Homeodomain-interacting protein kinase |
| ^a^ Potentially involved in transmembrane and intracellular transportation of endocytosis process  ^b^ *Dicyema*-specific expansion (Z-score calculated among compared bilaterians is larger than 2) | | |

| **Table S4. The most abundant domains in dicyemids compared with selected bilaterians** | | | | | | | | | | | | | | | |
| --- | --- | --- | --- | --- | --- | --- | --- | --- | --- | --- | --- | --- | --- | --- | --- |
| Pfam domain | Pfam ID | Function | dja | ili | emu | sma | sme | lgi | obi | cte | hro | dme | cel | bfl | hsa |
| Pkinase | PF00069 | Protein kinase domain | 142 | 171 | 245 | 245 | 725 | 322 | 389 | 379 | 474 | 447 | 562 | 556 | 482 |
| Pkinase_Tyr | PF07714 | Protein tyrosine kinase | 137 | 163 | 238 | 239 | 687 | 318 | 381 | 365 | 461 | 442 | 541 | 556 | 477 |
| WD40 | PF00400 | WD domain, G-beta repeat | 86 | 124 | 134 | 159 | 204 | 211 | 290 | 232 | 188 | 207 | 168 | 244 | 252 |
| RRM_1 | PF00076 | RNA recognition motif | 67 | 103 | 139 | 151 | 224 | 136 | 208 | 145 | 209 | 265 | 164 | 119 | 214 |
| Helicase_C | PF00271 | Helicase conserved C-terminal domain | 62 | 56 | 82 | 83 | 133 | 83 | 118 | 94 | 91 | 99 | 107 | 96 | 107 |
| ANAPC4_WD40 | PF12894 | Anaphase-promoting complex subunit 4 WD40 domain | 60 | 77 | 89 | 110 | 151 | 174 | 203 | 172 | 148 | 165 | 124 | 192 | 200 |
| Roc | PF08477 | Ras of Complex, Roc, domain of DAPkinase | 56 | 73 | 66 | 83 | 200 | 136 | 131 | 142 | 118 | 133 | 97 | 206 | 185 |
| Ras | PF00071 | Ras family | 55 | 68 | 65 | 78 | 180 | 137 | 134 | 134 | 119 | 127 | 93 | 191 | 178 |
| DEAD | PF00270 | DEAD/DEAH box helicase | 55 | 54 | 67 | 68 | 123 | 70 | 90 | 86 | 81 | 81 | 89 | 92 | 90 |
| TPR_2 | PF07719 | Tetratricopeptide repeat | 54 | 49 | 59 | 81 | 107 | 106 | 140 | 119 | 86 | 102 | 73 | 396 | 142 |
| Kinase-like | PF14531 | Kinase-like | 53 | 45 | 85 | 77 | 250 | 128 | 118 | 106 | 161 | 134 | 151 | 120 | 185 |
| Arf | PF00025 | ADP-ribosylation factor family | 50 | 65 | 63 | 82 | 187 | 123 | 115 | 126 | 104 | 114 | 103 | 139 | 180 |
| EF-hand_6 | PF13405 | EF-hand domain | 49 | 68 | 87 | 100 | 212 | 202 | 176 | 175 | 146 | 151 | 108 | 248 | 173 |
| EF-hand_7 | PF13499 | EF-hand domain pair | 48 | 69 | 90 | 104 | 213 | 198 | 176 | 182 | 142 | 152 | 112 | 250 | 179 |
| EF-hand_1 | PF00036 | EF hand | 48 | 68 | 85 | 103 | 213 | 207 | 181 | 184 | 144 | 156 | 116 | 252 | 187 |
| TPR_1 | PF00515 | Tetratricopeptide repeat | 44 | 40 | 49 | 67 | 100 | 101 | 124 | 105 | 78 | 88 | 64 | 379 | 128 |
| TPR_8 | PF13181 | Tetratricopeptide repeat | 43 | 34 | 46 | 60 | 92 | 86 | 108 | 94 | 66 | 82 | 55 | 358 | 114 |
| MMR_HSR1 | PF01926 | ribosome-binding GTPase | 43 | 74 | 68 | 91 | 245 | 160 | 127 | 146 | 128 | 156 | 122 | 204 | 195 |
| AAA_22 | PF13401 | AAA domain | 42 | 51 | 61 | 66 | 121 | 109 | 118 | 134 | 87 | 130 | 104 | 147 | 120 |
| AAA | PF00004 | ATPase family associated with various cellular activities (AAA) | 41 | 54 | 60 | 71 | 119 | 86 | 100 | 99 | 76 | 108 | 83 | 102 | 90 |
| EF-hand_8 | PF13833 | EF-hand domain pair | 40 | 56 | 67 | 83 | 154 | 143 | 121 | 142 | 117 | 123 | 83 | 194 | 140 |
| ResIII | PF04851 | Type III restriction enzyme, res subunit | 40 | 47 | 47 | 53 | 107 | 74 | 71 | 73 | 76 | 75 | 69 | 73 | 88 |
| TPR_12 | PF13424 | Tetratricopeptide repeat | 39 | 37 | 45 | 63 | 88 | 98 | 109 | 97 | 69 | 70 | 60 | 392 | 111 |
| LRR_4 | PF12799.5 | Leucine Rich repeats | 34 | 50 | 60 | 60 | 130 | 163 | 186 | 434 | 120 | 169 | 93 | 964 | 251 |
| AAA_5 | PF07728 | AAA domain (dynein-related subfamily) | 34 | 45 | 50 | 52 | 86 | 71 | 82 | 77 | 62 | 82 | 65 | 78 | 65 |
| TPR_19 | PF14559 | Tetratricopeptide repeat | 33 | 28 | 44 | 61 | 73 | 74 | 92 | 77 | 47 | 68 | 59 | 229 | 98 |
| AAA_16 | PF13191.4 | AAA ATPase domain | 33 | 44 | 58 | 66 | 120 | 101 | 106 | 166 | 73 | 135 | 98 | 134 | 129 |
| TPR_14 | PF13428.4 | Tetratricopeptide repeat | 31 | 22 | 46 | 67 | 59 | 79 | 91 | 81 | 55 | 67 | 60 | 314 | 108 |
| EF-hand_5 | PF13202.4 | EF hand | 30 | 50 | 65 | 72 | 144 | 158 | 121 | 133 | 113 | 107 | 83 | 211 | 120 |
| Ank_3 | PF13606.4 | Ankyrin repeat | 29 | 57 | 80 | 85 | 350 | 181 | 432 | 398 | 149 | 173 | 126 | 222 | 241 |
| Ank_2 | PF12796.5 | Ankyrin repeat | 29 | 67 | 90 | 94 | 391 | 198 | 453 | 418 | 164 | 184 | 137 | 237 | 258 |
| dja, *Dicyema japonicum*; ili, *Intoshia linei*; emu, *Echinococcus multilocularis*; sma, *Schistosoma mansoni*; lgi, *Lottia gigantea*; obi, *Octopus bimaculoides*; cte, *Capitella teleta*; hro, *Hellobdella robusta*; dme, *Drosophila melanogaster*; cel, *Caenorhabditis elegans*; bfl, *Branchiostoma floridae*; hsa, *Homo sapiens*. | | | | | | | | | | | | | | | |

| **Table S5. Numbers of genes with transcription factor-related domains in selected bilaterians** | | | | | | | | | | | | | | | |
| --- | --- | --- | --- | --- | --- | --- | --- | --- | --- | --- | --- | --- | --- | --- | --- |
| Pfam domain | Pfam ID | Function | dja | ili | emu | sma | sme | lgi | obi | cte | hro | dme | cel | bfl | hsa |
| Homeobox | PF00046 | Homeobox domain | 16 | 65 | 77 | 77 | 133 | 141 | 96 | 183 | 242 | 164 | 125 | 127 | 244 |
| zf-C2H2 | PF00096 | Zinc finger, C2H2 type | 13 | 42 | 97 | 99 | 138 | 331 | 2086 | 325 | 234 | 338 | 157 | 986 | 709 |
| HLH | PF00010 | Helix-loop-helix DNA-binding domain | 14 | 25 | 31 | 35 | 50 | 78 | 61 | 85 | 70 | 80 | 48 | 80 | 108 |
| HMG_box | PF00505 | HMG (high mobility group) box | 11 | 13 | 20 | 20 | 47 | 29 | 71 | 25 | 66 | 37 | 23 | 45 | 56 |
| Homeobox_KN | PF05920 | Homeobox KN domain | 10 | 19 | 34 | 27 | 49 | 50 | 27 | 66 | 115 | 42 | 41 | 40 | 79 |
| zf-C4 | PF00105 | Zinc finger, C4 type (two domains) | 8 | 12 | 15 | 21 | 32 | 36 | 33 | 38 | 50 | 47 | 325 | 29 | 46 |
| Ets | PF00178 | Ets-domain | 7 | 3 | 9 | 9 | 22 | 10 | 13 | 13 | 22 | 15 | 17 | 13 | 28 |
| bZIP_1 | PF00170 | bZIP transcription factor | 5 | 6 | 14 | 13 | 31 | 38 | 27 | 33 | 28 | 39 | 39 | 38 | 50 |
| Hormone_recep | PF00104 | Ligand-binding domain of nuclear hormone receptor | 4 | 10 | 13 | 14 | 15 | 32 | 35 | 38 | 32 | 42 | 320 | 29 | 48 |
| ARID | PF01388 | ARID/BRIGHT DNA binding domain | 3 | 4 | 6 | 7 | 8 | 6 | 14 | 7 | 9 | 9 | 6 | 4 | 15 |
| Pou | PF00157 | Pou domain - N-terminal to homeobox domain | 3 | 2 | 6 | 6 | 10 | 4 | 4 | 6 | 11 | 18 | 5 | 6 | 16 |
| bZIP_Maf | PF03131 | bZIP Maf transcription factor | 2 | 1 | 8 | 8 | 9 | 13 | 11 | 13 | 16 | 22 | 19 | 15 | 35 |
| Forkhead | PF00250 | Fork head domain | 2 | 21 | 16 | 16 | 33 | 31 | 19 | 47 | 31 | 27 | 32 | 31 | 50 |
| GATA | PF00320 | GATA zinc finger | 2 | 3 | 5 | 6 | 8 | 7 | 10 | 16 | 15 | 11 | 27 | 8 | 19 |
| RHD_DNA_bind | PF00554 | Rel homology DNA-binding domain | 2 | 0 | 1 | 0 | 4 | 4 | 5 | 3 | 4 | 12 | 0 | 2 | 10 |
| CUT | PF02376 | CUT domain | 1 | 2 | 3 | 4 | 7 | 3 | 2 | 3 | 11 | 6 | 6 | 3 | 7 |
| PAX | PF00292 | Paired box' domain | 1 | 5 | 4 | 4 | 14 | 8 | 6 | 9 | 10 | 20 | 12 | 5 | 9 |
| Runt | PF00853 | Runt domain | 1 | 0 | 1 | 1 | 2 | 1 | 1 | 2 | 2 | 8 | 1 | 1 | 3 |
| SRF-TF | PF00319 | SRF-type transcription factor (DNA- binding domain) | 1 | 2 | 3 | 4 | 5 | 3 | 3 | 2 | 6 | 3 | 2 | 4 | 5 |
| Basic | PF01586 | Myogenic Basic domain | 0 | 1 | 1 | 1 | 1 | 1 | 1 | 1 | 1 | 2 | 0 | 4 | 4 |
| DM | PF00751 | DM DNA binding domain | 0 | 1 | 2 | 4 | 6 | 4 | 2 | 5 | 3 | 4 | 13 | 10 | 7 |
| GCM | PF03615 | GCM motif protein | 0 | 1 | 2 | 2 | 2 | 1 | 1 | 1 | 2 | 3 | 0 | 2 | 2 |
| Hairy_orange | PF07527 | Hairy Orange | 0 | 1 | 0 | 1 | 1 | 18 | 5 | 11 | 2 | 12 | 0 | 13 | 11 |
| Neuro_bHLH | PF12533 | Neuronal helix-loop-helix transcription factor | 0 | 0 | 1 | 1 | 1 | 1 | 1 | 2 | 1 | 0 | 1 | 1 | 4 |
| OAR | PF03826 | OAR domain | 0 | 0 | 0 | 0 | 0 | 9 | 11 | 5 | 4 | 11 | 0 | 12 | 15 |
| P53 | PF00870 | P53 DNA-binding domain | 0 | 0 | 2 | 3 | 1 | 1 | 1 | 1 | 2 | 2 | 0 | 4 | 3 |
| HPD | PF05044 | Homeo-prospero domain | 0 | 1 | 2 | 2 | 4 | 1 | 1 | 1 | 3 | 1 | 1 | 0 | 2 |
| SCAN | PF02023 | SCAN domain | 0 | 0 | 0 | 0 | 0 | 2 | 3 | 0 | 2 | 0 | 0 | 0 | 60 |
| T-box | PF00907 | T-box | 0 | 7 | 5 | 7 | 5 | 12 | 12 | 8 | 18 | 14 | 21 | 11 | 17 |
| TF_AP-2 | PF03299 | Transcription factor AP-2 | 0 | 3 | 1 | 1 | 2 | 1 | 2 | 2 | 2 | 6 | 5 | 2 | 5 |
| TF_Otx | PF03529 | Otx1 transcription factor | 0 | 0 | 0 | 0 | 0 | 0 | 0 | 0 | 0 | 0 | 0 | 1 | 3 |
| zf-C2HC | PF01530 | Zinc finger, C2HC type | 0 | 0 | 2 | 2 | 5 | 3 | 6 | 5 | 3 | 7 | 2 | 2 | 7 |
| dja, *Dicyema japonicum*; ili, *Intoshia linei*; emu, *Echinococcus multilocularis*; sma, *Schistosoma mansoni*; lgi, *Lottia gigantea*; obi, *Octopus bimaculoides*; cte, *Capitella teleta*; hro, *Hellobdella robusta*; dme, *Drosophila melanogaster*; cel, *Caenorhabditis elegans*; bfl, *Branchiostoma floridae*; hsa, *Homo sapiens*. | | | | | | | | | | | | | | | |

| **Table S6. Numbers of genes with signaling pathway-related domains in selected bilaterian** | | | | | | | | | | | | | | | | |
| --- | --- | --- | --- | --- | --- | --- | --- | --- | --- | --- | --- | --- | --- | --- | --- | --- |
| Pfam domain | Pfam ID | Function | dja | ili | emu | sma | sme | lgi | obi | cte | hro | dme | cel | bfl | hsa |  |
| G-alpha | PF00503 | G-protein alpha subunit | 14 | 25 | 26 | 32 | 66 | 36 | 33 | 42 | 35 | 36 | 50 | 31 | 49 |  |
| RGS | PF00615 | Regulator of G protein signaling domain | 7 | 10 | 13 | 11 | 30 | 14 | 24 | 17 | 17 | 18 | 31 | 14 | 36 |  |
| EGF | PF00008 | EGF-like domain | 4 | 25 | 27 | 25 | 38 | 109 | 169 | 225 | 102 | 87 | 74 | 541 | 127 |  |
| DIX | PF00778 | DIX domain | 2 | 4 | 4 | 5 | 5 | 4 | 4 | 3 | 3 | 5 | 8 | 3 | 7 |  |
| TGF_beta | PF00019 | Transforming growth factor beta like domain | 2 | 4 | 3 | 2 | 11 | 11 | 14 | 14 | 8 | 7 | 5 | 20 | 37 |  |
| Notch | PF00066 | LNR domain | 1 | 0 | 2 | 1 | 3 | 2 | 3 | 6 | 3 | 3 | 2 | 3 | 6 |  |
| Frizzled | PF01534 | Frizzled/Smoothened family membrane region | 1 | 2 | 6 | 7 | 12 | 5 | 6 | 8 | 6 | 10 | 7 | 7 | 12 |  |
| STAT_bind | PF02864 | STAT protein, DNA binding domain | 1 | 0 | 0 | 0 | 2 | 2 | 2 | 6 | 3 | 1 | 5 | 4 | 7 |  |
| STAT_int | PF02865 | STAT protein, protein interaction domain | 1 | 0 | 0 | 0 | 0 | 1 | 2 | 6 | 2 | 1 | 0 | 3 | 7 |  |
| wnt | PF00110 | wnt family | 1 | 4 | 6 | 6 | 9 | 12 | 17 | 16 | 18 | 8 | 6 | 15 | 19 |  |
| CHRD | PF07452 | CHRD domain | 0 | 0 | 0 | 0 | 0 | 1 | 1 | 0 | 0 | 6 | 0 | 1 | 1 |  |
| Dishevelled | PF02377 | Dishevelled specific domain | 0 | 0 | 2 | 2 | 2 | 1 | 1 | 1 | 0 | 1 | 4 | 1 | 4 |  |
| DSL | PF01414 | Delta serrate ligand | 0 | 1 | 12 | 7 | 36 | 11 | 9 | 8 | 5 | 10 | 15 | 11 | 4 |  |
| FGF | PF00167 | Fibroblast growth factor | 0 | 1 | 0 | 0 | 1 | 2 | 4 | 1 | 1 | 4 | 2 | 9 | 25 |  |
| Focal_AT | PF03623 | Focal adhesion targeting region | 0 | 0 | 1 | 1 | 1 | 1 | 2 | 1 | 1 | 3 | 3 | 2 | 2 |  |
| G-gamma | PF00631 | GGL domain | 0 | 1 | 4 | 2 | 6 | 4 | 6 | 6 | 6 | 8 | 7 | 1 | 16 |  |
| HH_signal | PF01085 | Hedgehog amino-terminal signalling domain | 0 | 0 | 1 | 1 | 1 | 3 | 3 | 1 | 1 | 1 | 0 | 3 | 3 |  |
| MCPsignal | PF00015 | Methyl-accepting chemotaxis protein (MCP) signalling domain | 0 | 0 | 1 | 0 | 0 | 7 | 1 | 1 | 0 | 1 | 0 | 11 | 0 |  |
| PDGF | PF00341 | PDGF/VEGF domain | 0 | 0 | 0 | 0 | 0 | 2 | 2 | 2 | 1 | 6 | 1 | 2 | 9 |  |
| Phe_ZIP | PF08916 | Phenylalanine zipper | 0 | 0 | 0 | 0 | 0 | 0 | 0 | 0 | 0 | 1 | 0 | 0 | 3 |  |
| PTN_MK_C | PF01091 | PTN/MK heparin-binding protein family, C-terminal domain | 0 | 0 | 0 | 0 | 0 | 0 | 2 | 1 | 0 | 4 | 0 | 2 | 2 |  |
| PTN_MK_N | PF05196 | PTN/MK heparin-binding protein family, N-terminal domain | 0 | 0 | 0 | 0 | 0 | 1 | 1 | 0 | 1 | 0 | 0 | 1 | 2 |  |
| Rabaptin | PF03528 | Rabaptin | 0 | 0 | 0 | 1 | 1 | 1 | 1 | 1 | 1 | 0 | 0 | 2 | 2 |  |
| STAT_alpha | PF01017 | STAT protein, all-alpha domain | 0 | 0 | 0 | 0 | 0 | 1 | 0 | 5 | 1 | 1 | 1 | 3 | 7 |  |
| TGFb_propeptide | PF00688 | TGF-beta propeptide | 0 | 0 | 1 | 0 | 3 | 10 | 11 | 8 | 5 | 7 | 1 | 15 | 24 |  |
| dja, *Dicyema japonicum*; ili, *Intoshia linei*; emu, *Echinococcus multilocularis*; sma, *Schistosoma mansoni*; lgi, *Lottia gigantea*; obi, *Octopus bimaculoides*; cte, *Capitella teleta*; hro, *Hellobdella robusta*; dme, *Drosophila melanogaster*; cel, *Caenorhabditis elegans*; bfl, *Branchiostoma floridae*; hsa, *Homo sapiens*. | | | | | | | | | | | | | | | | |
