# Supplementary figures and images for: Dicyemid Mesozoans: A Unique Parasitic Lifestyle and a Reduced Genome
Source: Genome Biol Evol. 2019 Jul 26;11(8):2232–43. doi: 10.1093/gbe/evz157 (PMC6736024; doi:10.1093/gbe/evz157)

Figure S1.

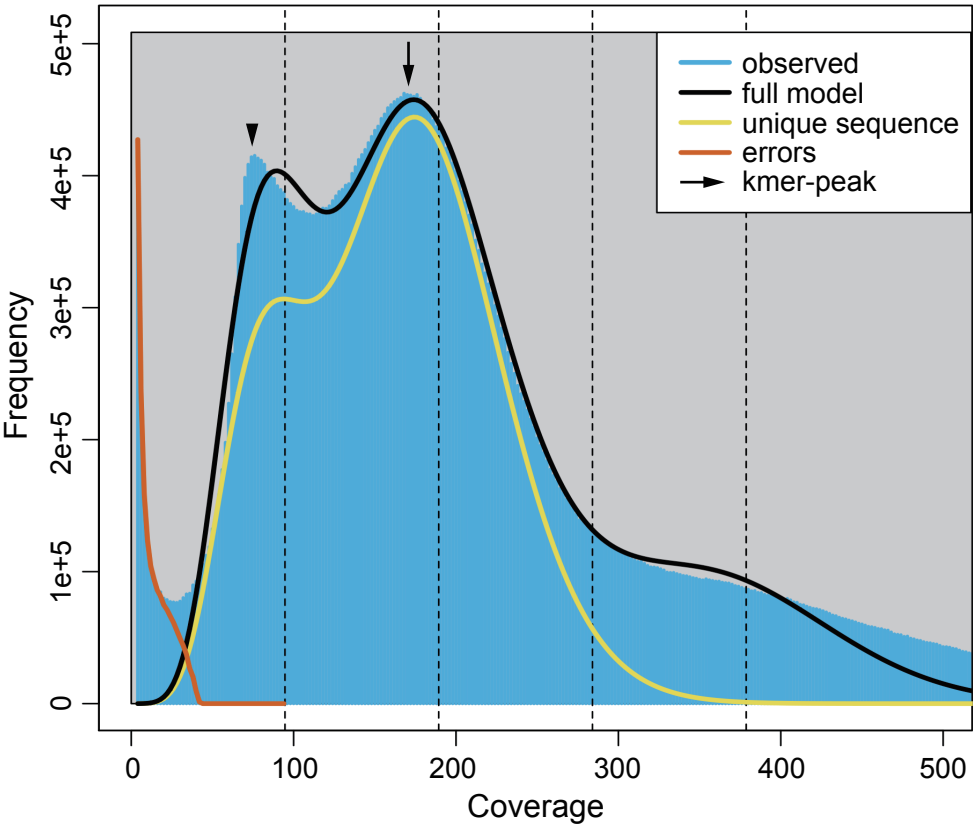

Supplement: evz157_Supplementary_Data [file evz157_supplementary_data.zip › Fig_S1.pdf]

Figure S2.

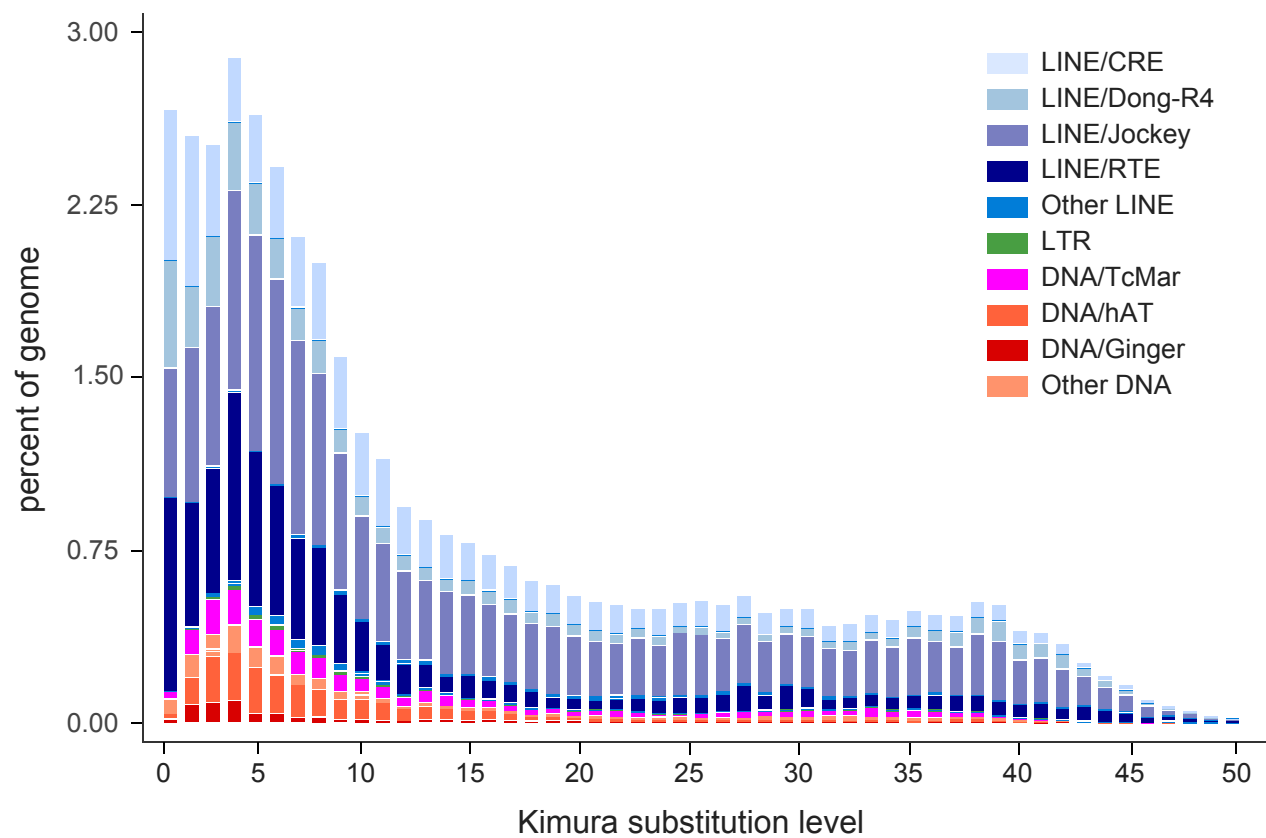

Supplement: evz157_Supplementary_Data [file evz157_supplementary_data.zip › Fig_S2.pdf]
